# Nbs1 ChIP-Seq Identifies Off-Target DNA Double-Strand Breaks Induced by AID in Activated Splenic B Cells

**DOI:** 10.1371/journal.pgen.1005438

**Published:** 2015-08-11

**Authors:** Lyne Khair, Richard E. Baker, Erin K. Linehan, Carol E. Schrader, Janet Stavnezer

**Affiliations:** Department of Microbiology and Physiological Systems, University of Massachusetts Medical School, Worcester, Massachusetts, United States of America; Duke University, UNITED STATES

## Abstract

Activation-induced cytidine deaminase (AID) is required for initiation of Ig class switch recombination (CSR) and somatic hypermutation (SHM) of antibody genes during immune responses. AID has also been shown to induce chromosomal translocations, mutations, and DNA double-strand breaks (DSBs) involving non-Ig genes in activated B cells. To determine what makes a DNA site a target for AID-induced DSBs, we identify off-target DSBs induced by AID by performing chromatin immunoprecipitation (ChIP) for Nbs1, a protein that binds DSBs, followed by deep sequencing (ChIP-Seq). We detect and characterize hundreds of off-target AID-dependent DSBs. Two types of tandem repeats are highly enriched within the Nbs1-binding sites: long CA repeats, which can form Z-DNA, and tandem pentamers containing the AID target hotspot WGCW. These tandem repeats are not nearly as enriched at AID-independent DSBs, which we also identified. Msh2, a component of the mismatch repair pathway and important for genome stability, increases off-target DSBs, similar to its effect on Ig switch region DSBs, which are required intermediates during CSR. Most of the off-target DSBs are two-ended, consistent with generation during G1 phase, similar to DSBs in Ig switch regions. However, a minority are one-ended, presumably due to conversion of single-strand breaks to DSBs during replication. One-ended DSBs are repaired by processes involving homologous recombination, including break-induced replication repair, which can lead to genome instability. Off-target DSBs, especially those present during S phase, can lead to chromosomal translocations, deletions and gene amplifications, resulting in the high frequency of B cell lymphomas derived from cells that express or have expressed AID.

## Author Summary

Activation-induced cytidine deaminase (AID) is required for diversifying antibodies during immune responses, and it does this by introducing mutations and DNA breaks into antibody genes. How AID is targeted is not understood, and it induces chromosomal translocations, mutations, and double-strand breaks (DSBs) at sites other than antibody genes in activated B cells. To determine what makes an off-target DNA site a target for AID-induced DSBs, we identify and characterize hundreds of genome-wide DSBs induced by AID during B cell activation. Interestingly, many of the DSBs are within or adjacent to two types of tandemly repeated simple sequences, which have characteristics that might explain why they are targeted. We find that most of the DSBs are two-ended, consistent with their generation during G1 phase of the cell cycle, which is when AID induces DNA breaks in antibody genes. However, a minority is one-ended, consistent with replication encountering an AID-induced single-strand break, thereby creating a DSB. Both types of off-target DSBs, but especially those present during S phase of the cell cycle, lead to chromosomal translocations, deletions and gene amplifications that can promote B cell lymphomagenesis.

## Introduction

Activation-induced cytidine deaminase (AID) is required for initiation of somatic hypermutation (SHM) of Ig variable region genes and class switch recombination (CSR) of IgH genes in B cells during an immune response [1,2]. Both SHM and CSR are required for effective humoral immune responses, and thus humans (and mice) lacking AID are severely immunocompromised. AID deaminates cytosines (dC) in expressed Ig variable region genes and in IgH switch (S) regions, converting dC to uracil (dU), which can then be replicated by DNA polymerase (Pol) to form dC>dT mutations. Alternatively, the dU base is excised by uracil DNA glycosylase (primarily Ung), which leaves an abasic, or apyrimidinic/apurinic (AP) site [3,4]. AP sites cannot be copied by high-fidelity DNA Pol, but can serve as templates for error-prone translesion DNA Pols, which insert any base across from the AP site. Alternatively, AP sites are incised by AP-endonucleases (Ape1/Ape2, also termed Apex1/Apex2) to create single-strand DNA breaks (SSBs). If SSBs on opposite strands are sufficiently near each other, they form a double-strand break (DSB). If they are farther apart, they can still generate DSBs with the help of the mismatch repair (MMR) system, after recognition of a dU:dG mismatch by Msh2-Msh6, followed by excision of one strand from a nick created by Ape1/2 [5]. During CSR, AID-dependent DSBs are induced within IgH S regions, which are highly enriched in the AID target hotspot, WGCW, in which W is A or T, and the C on both strands is a hotspot target, thus increasing the probability of AID-induced SSBs leading to DSBs.

For unknown reasons, AID acts predominantly on Ig genes in activated B cells, although it can act at other sites in the genome with reduced frequency. This was first demonstrated by the finding of AID-dependent mutations in several actively transcribed non-Ig genes in germinal center B cells, where AID is highly expressed and SHM of Ig genes occurs [6–11]. In addition, AID has been demonstrated to instigate off-target DSBs and chromosomal translocations in B cells induced to undergo CSR in culture [12–22]. Chromosomal deletions, duplications, and translocations are found in human B cell lymphomas and gastric and prostate cancers, many of which might be instigated by AID [23–25]; thus, it is important to understand what causes non-Ig chromosomal sites to become susceptible to AID-dependent DSBs. Furthermore, what causes some off-target sites mutated by AID to progress to DSBs is unknown.

Genome-wide AID-dependent DSBs have been detected in mouse splenic B cells undergoing CSR by using Nbs1-ChIP followed by hybridization to tiling arrays of the entire genome (ChIP-chip) [15]. Nbs1 has been shown to bind AID-dependent DSBs, most strongly at the IgH Sμ region, which is the upstream/donor S region for most CSR events [15,26]. CSR occurs by non-homologous end-joining (NHEJ) in the G1 phase of the cell cycle [5]. Consistent with this, Ku70-Ku80 and DNA-PKcs bind to S region DSBs, and cells deficient in these NHEJ proteins show reduced CSR [27,28]. Recent results suggest that during CSR, blunt or nearly blunt DSBs are recombined by NHEJ, but those with longer 3’ ss tails recombine using micro-homology-mediated end-joining, also termed alternative-end joining (A-EJ) [29]. The Mre11-Rad50-Nbs1 (MRN) complex and CtIP are important for end-resection during A-EJ, which also occurs during G1 phase [29–34]. Ku binding at DSBs is transient, as Ku slides away from DSB ends [35], and Ku80 is rapidly ubiquitinated by RNF8 [36]. MRN could subsequently bind DNA ends that are not rapidly recombined by NHEJ, perhaps because they do not have the correct blunt structure. A-EJ, rather than NHEJ, has been shown to be involved in AID-dependent chromosomal translocations in mouse cells [37–39]. Homologous recombination in G2 phase cells also involves MRN, with more extensive end-resection by CtIP [40,41]. By using Nbs1 ChIP, our screen could be biased towards detecting off-target DSBs that are not immediately repaired/recombined, and are therefore capable of causing genomic instability.

In this study, we identify off-target AID-dependent DSBs in mouse splenic B cells induced to switch in culture using Nbs1 ChIP-Seq, as this allows a more precise determination of the Nbs1-binding sites than does ChIP-chip. The Nbs1-binding sites separate into different classes, 66–70% are within genes/regions transcribed by RNA polymerase II (Pol II), many contain tandem repeats of the AID hotspot target motif, WGCW, and others have tandem CA repeats but very few AID hotspots, and most are two-ended DSBs but a minority are one-ended, indicating they were generated by replication. Our data suggest that whether an AID-induced deamination progresses to a SSB, and then on to a DSB, is highly dependent upon its sequence context, and we have identified sites where AID-induced mutations are prone to generate DSBs.

## Results and Discussion

**Fig 1:**
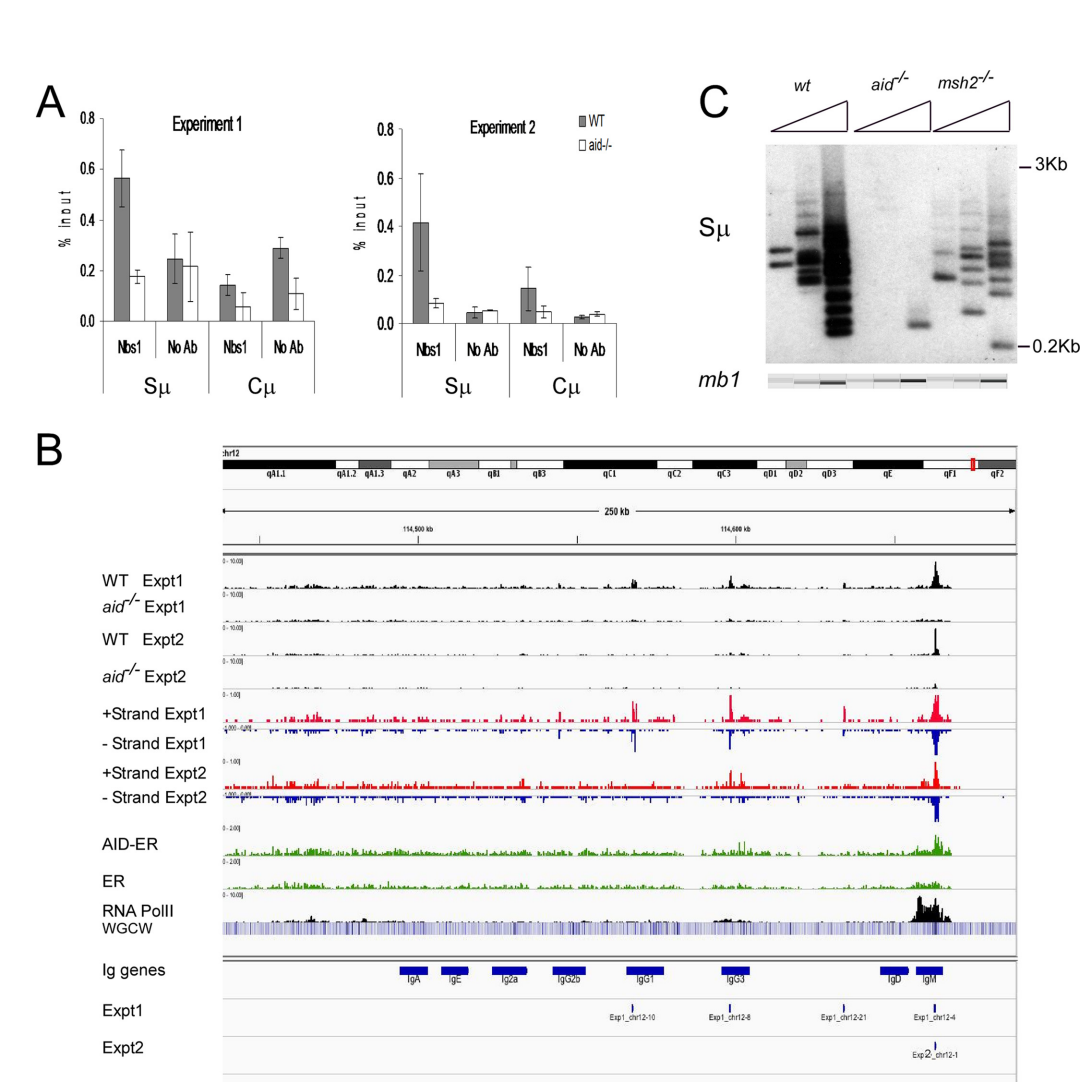
Nbs1 binds at IgH Sμ in cultured mouse splenic B cells induced to undergo CSR. **A**: Quantitative PCR analysis of Nbs1 ChIP material used for deep sequencing. Mean % input +/- SEM are shown. **B**: Browser tracks for Nbs1 ChIP at the IgH locus in WT and *aid* ^*-/-*^ cells in both experiments (plotted as numbers of aligned tags per million sequences) along with tracks showing the plus (red) and minus (blue) strand coverage for the WT samples. Below that are coverage tracks (green) for AID-ER and control ER ChIP’s and for Pol II binding (black). Below this, a heat map shows the concentration of WGCW sites, allowing the localization of IgH S regions. Below this are the locations of the 8 Ig C_H_ region genes (including I exons and S regions), labeled “IgM”, etc. Below the gene annotations are two bars indicating the Nbs1-binding sites called in Exp 1 and Exp 2. Each called site is mapped as a 0.5 kb segment. **C**: Ligation-mediated PCR (LM-PCR) analysis shows DSBs detected at Sμ in WT, *aid* ^*-/-*^ and *msh2* ^*-/-*^ cells. In all LM-PCR assays in this report, 3-fold titrations of template DNA were used, and the *mb-1* gene was amplified as an internal control for template input, and assayed using microfluidics (QIAxcel Advanced instrument).

To detect AID-dependent off-target DSBs, we performed two independent experiments in which we cultured wild-type (WT) and *aid* ^*-/-*^ splenic B cells for two days under conditions that induce CSR to IgG3 (LPS+anti-IgD dextran), and performed ChIP-Seq using antibody to Nbs1. Two days of culture is optimal for detecting AID-dependent DSBs in S regions [42], and is the same timepoint used previously to identify genome-wide AID-dependent DSBs by Nbs1 ChIP-chip [15]. The immunoprecipitated samples were first evaluated by quantitative PCR (Fig 1A), which shows that Nbs1 binds to Sμ but not Cμ in WT B cells induced to switch, and does not bind to Sμ or Cμ in *aid* ^*-/-*^ cells. Immmunoprecipitated DNA was prepared for Illumina deep sequencing, and the sequences aligned to the mouse genome. The findPeaks program from the Homer suite [43] was used to identify regions enriched in the WT ChIP relative to the *aid* ^*-/-*^ ChIP, and an ad hoc filtering scheme was applied to eliminate peaks with low tag numbers and/or low WT: *aid* ^*-/-*^ enrichment. 801 and 284 AID-dependent Nbs1-binding sites were identified in experiments (Exps) 1 and 2, respectively (S1 and S2 Tables). Of these sites, 37 were identified in both experiments, termed reproducible AID-dependent sites (S3 Table). The variation between cultures is likely an indication of the transient nature of these DSBs, and that each experiment captures only a subset of genome-wide AID-induced DSBs. Variance could be caused by differences in AID targeting, differences among cells at any of the subsequent steps required to generate DSBs, and to unknown experimental differences. We also identified 28 reproducible AID-independent Nbs1-binding sites that had similar numbers of tags in both WT and *aid* ^*-/-*^ cells in both experiments (S4 Table).

To compare the Nbs1-binding sites with AID-binding sites under our experimental conditions, we transduced activated *aid* ^*-/-*^ splenic B cells with the retrovirus pMX-PIE-AID-ER [44]. This retrovirus expresses AID with a C terminal estrogen receptor (ER) tag, allowing us to enforce nuclear expression by treatment with tamoxifen, immunoprecipitate with anti-ER antibody, and use ChIP-Seq to detect AID binding in the genome. *Aid* ^*-/-*^ cells transduced with retrovirus expressing the ER tag alone served as control. To determine if the Nbs1-binding sites were located in regions transcribed by RNA Pol II, we also performed ChIP-seq for Pol II, in both WT and *aid* ^*-/-*^ cells. We found no difference in the patterns of Pol II binding between WT and *aid* ^*-/-*^ cells, except at the AID gene itself.

Fig 1B shows browser tracks for Nbs1 binding detected at the IgH locus in WT and *aid* ^*-/-*^ cells in both experiments along with tracks showing the plus (red) and minus (blue) strand coverage in WT cells for each experiment. Also shown are AID-ER binding, Pol II binding, and a heat map showing the concentration of WGCW sites, allowing the localization of IgH S regions. Below the gene annotations are bars indicating the Nbs1-binding sites identified in Exps 1 and 2. As expected, the Sμ region has a strong Nbs1-binding signal, with enrichments of 26- and 6-fold in WT cells relative to *aid* ^*-/-*^ cells, in Exps1 and 2, respectively. Fig 1C shows a representative ligation-mediated (LM)-PCR experiment to demonstrate DSBs in the Sμ region in cells activated identically as for ChIP-Seq experiments. This assay shows that Sμ DSBs are AID-dependent and are also decreased in *msh2* ^*-/-*^ cells, as previously reported [42,45]. Msh2-deficiency does not decrease cell proliferation or increase cell death in these cultures. Note that although there appears to be an AID-dependent Nbs1 signal at Sγ3, the signal is below the Homer peak-calling threshold. The low signal at Sγ3 is consistent with the hypothesis that DSBs at acceptor S regions are limiting for CSR [46,47], and thus they rapidly undergo recombination with Sμ and do not persist. In fact, there are fewer AID-dependent aligned tags at Sγ3 than at several off-target sites in the genome.

Binding of AID-ER relative to the ER background is detected across the Sμ and Sγ3 regions, and there is also some binding above background at other sites in the IgH locus shown in Fig 1B. Over-expressed AID has been reported to bind at thousands of sites in ChIP-Seq experiments in activated splenic B cells [16], but we detect little binding of AID-ER at other sites across the genome. In our experiments, AID-ER is not over-expressed, but instead expressed at levels equivalent to endogenous AID. (We determined this by quantitative RT-PCR using equally efficient primers specific for mRNA for endogenous AID or transduced AID-ER [48].) Also, AID binding to DNA might be only transient [49].

RNA Pol II binding is robust across the entire Iμ-Sμ-Cμ gene (labeled IgM in Fig 1B), starting upstream of the mapped gene, as expected because these cells are transcribing μ mRNA and μ germline transcripts. As previously reported, Pol II pauses and accumulates at Sμ [50,51]. We also observed an accumulation of Pol II at the 3’ end of the Cμ gene, likely due to pausing during transcription termination [52]. Pol II binding is much weaker across the Iγ3-Cγ3 gene, consistent with the fact that the rate of transcription of γ3 germline transcripts is much less than that of μ RNA in activated IgM+ B cells.

### Off-target AID-dependent Nbs1-binding sites correspond to AID-dependent DSBs

**Fig 2:**
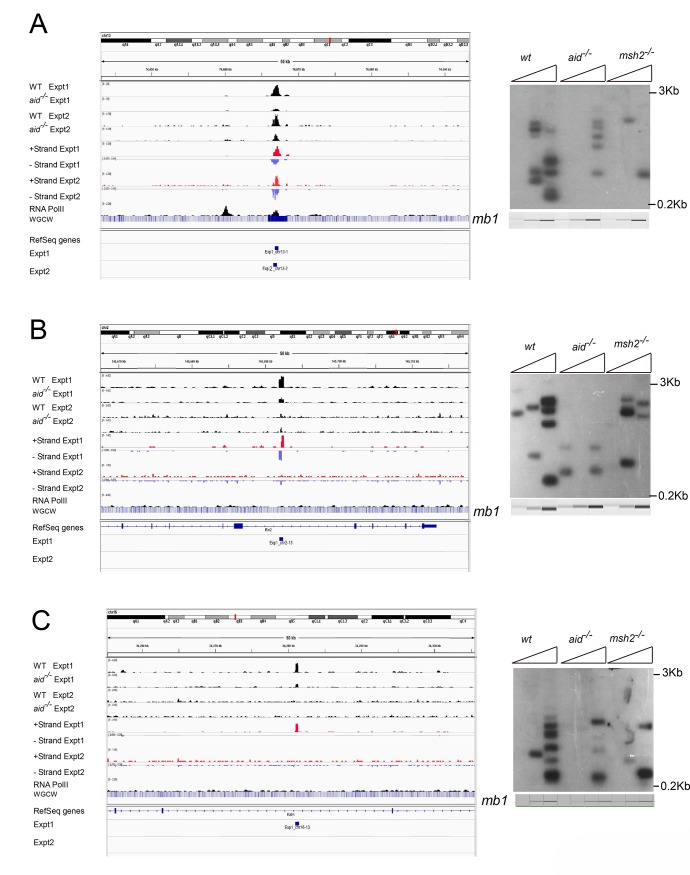
Three off target AID-dependent DSBs: browser tracks and LM-PCRs. **A:** Reproducible AID-dependent site on chromosome 3. **B, C:** Sites on chromsomes 2 and 16, respectively, called in Exp 1 only. Panel C shows an example of a one-ended DSB. S1–S4 Figs present additional examples of browser tracks and LM-PCR results for off-target AID-dependent DSBs.

To verify that the off-target AID-dependent Nbs1-binding sites are located at AID-dependent DSBs, we performed LM-PCR for several of the sites, using activated B cell DNA from two or more biologically independent experiments. We examined 6 of the 37 reproducible sites and 7 that were detected only in Exp 1. Eleven of these 13 Nbs1-binding sites showed AID-dependent DSBs in at least two independent experiments (Figs 2 and S1–S4; S1–S3 Tables). The cultures used for the LM-PCR experiments were independent of those used for the Nbs1 ChIP-seq experiments, suggesting that most of the AID-dependent DSBs are reproducible, despite the fact that they were not detected by Nbs1-ChIP in both experiments. Although Ig Sμ DSBs are detected reproducibly by LM-PCR in populations of B cells undergoing CSR, 50–150 cell-equivalents of genomic DNA are required to detect one Sμ DSB, suggesting they are present in only a small proportion of the cells at any one time [45,46]. Sμ DSBs are reproducibly detected in our ChIP-chip and ChIP-Seq experiments, including a few experiments that we do not include in this report. The weaker Nbs1 signals and fewer DSBs detected in LM-PCR assays of the off-target sites, relative to Sμ indicate that off-target DSBs are much less frequent. To detect one Sγ3 DSB in switching cells in our LM-PCR requires approximately 350–1100 cell-equivalents of genomic DNA. As Sγ3 DSBs are at the borderline of detection by Nbs1 ChIP-Seq, this suggests that the reproducible off-target DSBs are present in a somewhat greater proportion of cells than Sγ3 DSBs at any one moment. This low frequency could explain why two of the 13 Nbs1-binding sites tested by LM-PCR assay did not show AID-dependent DSBs.

**Fig 3:**
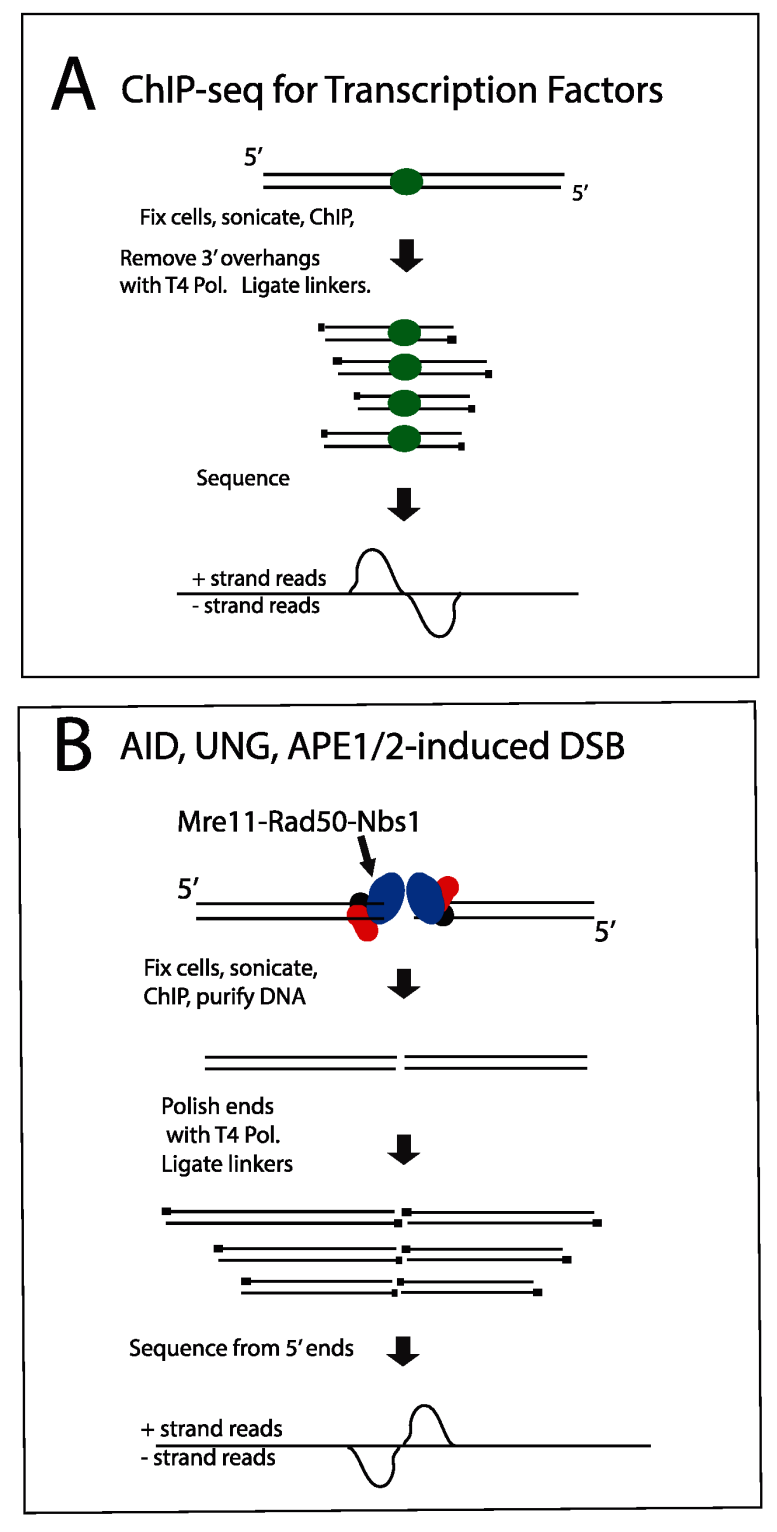
Diagram explaining orientation of +/- strand tags in ChIP-Seq. **A**: **Transcription factors.** After sonication and ChIP for a transcription factor, DNA ends are polished with T4 Pol, and linkers are ligated to the 5’ ends of the fragments. Sequencing initiates at the primers, resulting in plus strand sequences to the left/upstream of the transcription factor binding sites, and minus strand sequences on the right side of the binding sites. **B: DSBs.** The aligned sequences will pile up at the DSB, whereas the break due to sonication will be variable in position. Thus, when the sequences are aligned with the genome, the DSB position will be prominent relative to the sonicated ends. In this case, sequences obtained from the DSB end will correspond to the minus strand to the left of the DSB and correspond to the plus strand to the right of the DSB. If there were extensive resection at the DSB, the two peaks on the opposite strands would be separated from each other, but we do not observe this.

Examining strand specificity of the aligned tags provided further evidence that Nbs1 binding sites correspond to DSBs. Note that in the browser tracks of off-target sites shown in Fig 2A and 2B, the minus strand tags are located to the left of the plus strand tags. This is different from what is observed in ChIP-Seq data for transcription factors, where the plus strand tags are located to the left of the minus strand tags, as diagrammed in Fig 3A. In contrast, ChIP for proteins that bind at either side of a DSB should lead to the pattern observed in Figs 2A, 2B, S1 and S2, as diagrammed in Fig 3B and further explained in the figure legend. This pattern is reproducibly found at nearly all AID-dependent binding sites, unless there is a broad peak of Nbs1-binding, indicating numerous DSBs, which obscures this pattern (S3 and S4 Figs). This asymmetric pattern was also seen in most of the reproducible AID-independent sites, indicating these are also true DSBs (browser views available in the GEO database accession #GSE66424). The LM-PCR results and the strand-specific positions of the aligned tags relative to the called Nbs1 peaks indicate that most of the Nbs1-binding sites are indeed DSBs.

### One-ended DSBs

AID-dependent Sμ DSBs are generated and repaired/recombined during G1 phase [26,45,46]. Interestingly, ~6% of the AID-dependent DSBs (Table 1; example shown in Fig 2C) have tags that align on only one of the two strands, consistent with the pattern expected if the DSB is one-ended, as would be generated when DNA Pol encounters a SSB during replication. As a comparison, we performed the same analysis for Pol II binding sites and found less than 1 in 10^4^ sites have similarly skewed tags (S5 Fig). The one-ended DSBs are probably generated during S phase, suggesting that a small portion of off-target AID-dependent DSBs form when a SSB enters S phase. AID-dependent SSBs should rarely be introduced during S phase as Ung activity is restricted to G1 phase in activated B cells [53]. Two of the 4 one-ended reproducible AID-dependent DSBs are one-ended in only one of the two experiments. This suggests that some AID-dependent lesions can become DSBs within G1 phase in some cells, or be converted to DSBs by replicative Pol in other cells. DSBs generated by DNA Pol encountering a SSB would cause the replication fork to arrest. One-ended DSBs are usually repaired by homology-directed repair, explaining why B cells treated with an inhibitor of RAD51 or deficient in XRCC2, a protein important for homologous recombination, show unrepaired off-target AID-dependent DSBs [14,54,55]. Break-induced replication, a type of homologous recombination, is often used to repair one-ended DSBs, and this can lead to duplications, deletions, and inversions [56]. When homologous recombination is impaired, NHEJ might attempt to repair the one-ended DSB, and this can also result in gross chromosomal rearrangements [57].

**Table 1:** Characteristics of Nbs-1 binding sites and intersections with other sites.

	Number of Nbs1-binding sites	Total bp	Mean length (bp)	One-ended DSBs^a^ (% of sites)	Pol II at site or within gene (% of sites)	AID-binding at site [16]	Spt5 binding at site [10]	AID-dependent translocations at site (N) [17]	Within super-enhancer (N) [20]	xTSS at site (N) [78]	Nbs1 ChIP-chip (N) [15]
**Reproducible AID-dep. sites**	37	79,144	2139	10.8% (2 of 4 only in 1 exp)	70% *<0*.*001* ^b^	24% *<0*.*001*	13.5% *<0*.*001*	0	2.7% (1) *<0*.*001*	0	32.4% (12) *<0*.*001*
**AID-dep. sites (Exp 1)**	801	1,601,038	1999	8.1%	67% *<0*.*001*	26% *<0*.*001*	28% *<0*.*001*	1.0% (8) *<0*.*001*	3.1% (25) *<0*.*001*	4.5% (33) <0.001	12.2% (98) *0*.*001*
**AID-dep. sites (Exp 2)**	284	568,000	2000	1.1%	66% *<0*.*001*	27% *<0*.*001*	32%*<0*.*001*	0	2.8% (8) *<0*.*001*	10.6% (18) <0.001	20.0% (57) <*0*.*001*
**Reproducible AID-indep. sites**	28	63,734	2276	0	100% *<0*.*001*	64% *<0*.*001*	50% *<0*.*001*	0	7.1% (2) *0*.*002*	3.6% (1) *0*.*014*	25.0% *<0*.*001*

^a^ Intervals in which the tags on one strands are >2.8 fold greater than tags on the other strand. S5 Fig presents a graph of these ratios along with a graph of the Pol II control.

^b^ *p values* relative to random intervals as determined by USeq

### Similar to S region DSBs, off-target DSBs are decreased in *msh2* ^*-/-*^ cells

Canonical MMR is important for correcting mutations introduced during DNA replication in S phase. However, MMR is also important for formation of Ig Sμ DSBs in G1 phase, as Sμ DSBs are decreased by 50–80% in MMR-deficient B cells [45,58–60]. MMR is especially important for generating DSBs in Ig switch regions where the AID hotspot target sequence is not abundant, such as when the Sμ tandem repeat region has been deleted [58]. We asked if off-target AID-dependent DSBs are also dependent upon MMR in LM-PCR experiments using genomic DNA from *msh2* ^*-/-*^ cells, and found that all of the AID-dependent DSBs analyzed are reduced in frequency in Msh2-deficient cells (Figs 2 and S2–S4). Although Msh2 primarily protects against human B cell lymphoma [60–62], our data suggest that, in some cases, Msh2 might contribute to DSBs that could be associated with lymphomas initiated by AID activity. Msh2-deficient mice have been reported to have increased T cell but not B cell lymphomas, although Msh6-deficient mice develop both B and T cell lymphomas [63,64].

### Most AID-dependent DSBs are within transcribed genes or transcribed intergenic sites

Table 1 summarizes additional characteristics of the 37 reproducible AID-dependent Nbs1-binding sites, the AID-dependent DSBs detected in Exps 1 and 2, and reproducible AID-independent sites. For these analyses, the Nbs1 site called was extended by 1 kb on both sides of the peak center. This was done because Nbs1 has been shown by ChIP to bind within 1 kb of a defined DSB [65]. AID only targets Ig genes that are transcriptionally active, and in AID ChIP-Seq experiments performed in B cells induced to switch, the off-target AID-binding sites were mostly in transcribed genes [16]. As shown in Table 1, 70% of the reproducible AID-dependent Nbs1 binding sites and almost as many of the AID-dependent sites in the individual experiments are transcribed, as evidenced by the binding of Pol II at the site or within the gene in which the site is located. This result is similar to that obtained in the Nbs1 ChIP-chip study [15]. Note that some of the sites that bind Pol II are not in annotated genes (for example, Fig 2A). Interestingly, all of the reproducible AID-independent Nbs1 binding sites have Pol II binding (Table 1), indicating that transcriptionally active regions are prone to DSBs. It is possible that the 30% of AID-dependent sites that do not have detectable Pol II binding have very low levels of transcription or are transcribed by RNA Pols I or III, although we cannot rule out the possibility that ssDNA, the substrate for AID can be generated by means other than transcription, as discussed below.

### Tandem repeats are enriched at AID-dependent sites

**Fig 4:**
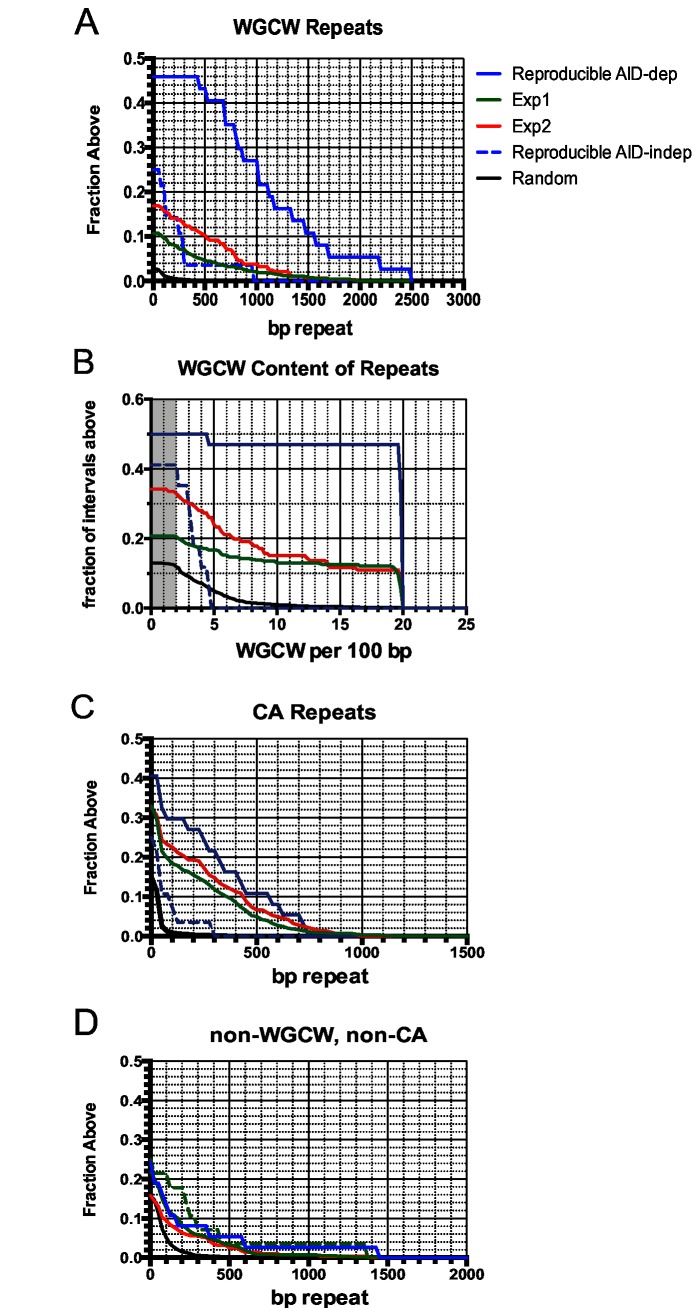
Accumulation plots indicate that specific tandem repeats are highly enriched at AID-dependent Nbs1 sites. **A:** Accumulation plot showing the proportion of the 37 reproducible AID-dependent DSB sites, and 801 sites detected in Exp1 and 284 sites detected in Exp 2 that have the indicated lengths of tandem repeats of WGCW motifs with a score of ≥100 in Tandem Repeat Finder. Also shown are lengths of WGCW repeats in the 28 reproducible AID-independent sites and 10,000 random intervals of the same length and chromosome distribution as the Nbs1-binding sites. **B:** Accumulation plot shows the density of the WGCW motifs in the indicated sets of Nbs1-binding sites. Sμ has a density of 19 repeats per 100 bp. Gray area indicates total genome average of WGCW is 2.0 per 100 bp. **C:** Accumulation plot to indicate the fraction of sites containing tandem CA repeats with a score of ≥60 in Tandem Repeat Finder. **D:** Accumulation plot indicates the fraction of sites in each pool that contain non-WGCW, non-CA repeats.

The reproducible AID-dependent Nbs1-binding sites are highly enriched in tandem repeats of WGCW, the AID hotspot target, relative to reproducible AID-independent sites and random sequences of the same lengths and chromosome distributions (Table 2; Fig 4). In fact, 46% of the AID-dependent off-target reproducible sites contain WGCW repeats that are at least 400 bp in length (Fig 4A). Although this motif is found at some of the reproducible AID-independent Nbs1 sites, they are fewer and the lengths of the repeats much shorter (median values: 1000 bp vs 100 bp, for reproducible AID-dependent and–independent sites, respectively). Also remarkable is that the density of the WGCW repeats (WGCW motifs per 100 bp) is much greater in AID-dependent sites than in the AID-independent sites (Fig 4B). As a comparison, in Sμ there are 19 WGCW motifs per 100 bp, and this same density is present in 43% of the reproducible off-target AID-dependent sites. In the off-target sites, the motif is a 5 bp motif, just as in Sμ, although the most common sequence of the motif is CAGCA, slightly different from Sμ, where it is GAGCT. As these motifs create AID target hotspots on both strands, this provides an attractive explanation for why reproducible AID-dependent DSBs are found at these tandem repeats.

**Table 2:** Tandem repeats at Nbs-1 binding sites.

	Number of Nbs1-binding sites	Total bp	Mean length (bp)	WGCW repeats^b^ (% of sites)	CA repeats^c^ (% of sites)	Either WGCW or CA repeats^b^ ^,^ ^c^ (% of sites)
**Reproducible AID-dep. sites** ^**a**^	37	79,144	2139	46%	30%	75.7%
**AID-dep. sites (Exp 1)**	801	1,601,038	1999	6%	18.6%	24.1%
**AID-dep. sites (Exp 2)**	284	568,000	2000	12%	22.5%	32.7%
**Reproducible AID-indep. sites**	28	63,734	2276	4%	7.1%	10.7%
**Random intervals**	10,000	2 x 10^7^	2000	0.23%^d^	0.97%^e^	1.19%

^a^ Reproducible sites are produced by merging overlapping sites for 2 experiments, explaining their greater length

^b^≥400 bp in length within called site

^c^≥100 bp in length within called site

^d^ 2,753 of 1.20 x 10^6^ 2-kb intervals in the genome (autosomes) have WGCW repeats ≥400 bp in length

^e^ 11,610 of 1.20 x 10^6^ 2-kb intervals in the genome (autosomes) have CA repeats ≥100 bp in length

About one-third of the reproducible AID-dependent DSBs contain a different tandem repeat, CA repeats at least 100 bp in length. The frequency of CA repeats at these sites is highly increased relative to that in random sequences (30% vs 1%) (Fig 4C) (Table 2). The median length of the repeats in reproducible AID-dependent sites is ~315 bp. CA repeats (≥100 bp in length) are also found at AID-independent Nbs1-binding sites, although much less frequently (7% of the sites). CA repeats greater than 30 bp in length can form unstable Z-DNA, a left-handed helix [66]. Due to the instability of this Z-DNA, it transitions between Z and B DNA; during the transition ss DNA might be accessible to AID. In addition, two bases are extruded from the helix at the junctions of Z and B DNA [67,68]. It is possible that CA repeats form ss DNA targets for AID, leading to SSBs, which are converted to DSBs by nuclease specific for structurally aberrant DNA, or perhaps during attempts to repair AID-induced lesions. Although CA repeats can lead to replication errors, this does not seem likely to explain their role in creating off-target AID-dependent DSBs since Ung activity, which is essential for nearly all AID-dependent SSBs and DSBs, is limited to G1 phase in activated B cells [53]. Other types of repeats, besides WGCW and CA, are not significantly enriched in the AID-dependent sites relative to AID-independent sites (Fig 4D). Also, at the reproducible AID-dependent sites there is no enrichment of inverted repeats, although they have been shown to cause genomic instability [69].

### Correspondence with Nbs1 ChIP-chip sites

Although only a few (4) of the AID-dependent Nbs1 ChIP-Seq sites correspond with the *reproducible* AID-dependent Nbs1-binding sites previously detected by ChIP-chip [15], a high proportion of the AID-dependent ChIP-Seq sites were identified as AID-dependent sites in one of the two ChIP-chip experiments (Table 1). To make this comparison we chose the ChIP-chip experiment with the higher signal-to-noise ratio and a total of 54,976 AID-dependent peaks called by NimbleScan Find-Peaks (Roche). The NimbleScan peak calls showed better correspondence with the AID-dependent ChIP-Seq sites than those produced by the Tamalpais peak caller used in ref [15]. Of the reproducible AID-dependent ChIP-Seq sites, 32% coincided with AID-dependent sites in the ChIP-chip experiment (Table 1). Two examples of intersecting sites are shown in S6 and S7 Figs. The AID-dependent ChIP-chip sites originally reported were also highly enriched in CA repeats and WGCW motifs [15]. Although the correspondence between the ChIP-Seq and ChIP-chip results is high, it is clear that our Nbs1-ChIP libraries are not saturated. As shown in Table 1, a significant portion of the AID-independent sites identified by ChIP-Seq also intersected with the AID-dependent ChIP-chip sites, suggesting that some of the AID-independent sites identified by ChIP-Seq might actually be weak AID targets. However, as a group the AID-independent sites have different properties from the AID-dependent sites, as discussed above.

### Comparisons of AID-dependent Nbs1-binding sites with results from other genome-wide studies

Approximately 25% of the AID-dependent DSBs correspond to previously-identified AID-binding sites in cells induced to switch with LPS+IL-4 [16], and the correspondence is highly significant compared with random sequences (Table 1). Surprisingly, the reproducible AID-independent sites show an even higher correlation with AID-binding than AID-dependent sites, perhaps because the AID-independent breaks are all found at Pol II binding sites or in genes with Pol II-binding sites, and because ChIP favors transcriptionally active accessible chromatin regions. AID interacts with Spt5, a factor associated with paused RNA Pol II, and Spt5 is thought to be important for recruiting AID to the genome [10]. Thousands of Spt5 binding sites have been identified by ChIP-Seq in B cells induced to switch with LPS+IL-4, and we compared the Nbs1-binding sites with these. About 29% of the AID-dependent DSBs occur at Spt5-binding sites, a highly significant correspondence (Table 1). However, 50% of AID-independent DSBs also occur at Spt5-binding sites.

Off-target AID-dependent DSBs can lead to chromosomal deletions, duplications, or translocations. Thus, we compared the Nbs1-binding sites with 234 AID-dependent translocation hotspots as defined by DNA regions that translocate to introduced I-Sce1 sites near *IgH* Sμ or within the *c-myc* locus in cells activated with LPS+IL-4 and over-expressing AID [17]. A small proportion (8) of the AID-dependent DSBs we identified occur at these AID-dependent translocation hotspots, but this is highly significant (Table 1). In a different study [20], 51 hotspots of AID-dependent translocation events with an I-Sce1 site introduced into *c-myc* were identified in anti-CD40+IL-4 activated B cells, but none of these sites are present among our AID-dependent Nbs1-binding sites. Possible explanations for why our AID-dependent DSB sites do not overlap at a higher frequency with translocation sites are: our Nbs1-ChIP library is not saturated; differences in activation methods (+/- IL-4), their use of over-expressed AID [17], and the DSBs we identify might be involved in translocations with sites other than *IgH* or *c-myc*. Also, it is possible that Nbs1-ChIP preferentially detects off-target DSBs that are slowly repaired or recombined. It is likely that the AID-dependent translocation hotspots identified in these studies [17,20] are within regions sufficiently near the *IgH* or *c-myc* loci to be able to recombine with them at a high frequency [70]. This possibility is consistent with the very low Nbs1 signals detected at Sγ3 in cells undergoing active IgG3 CSR. We hypothesize that Sγ3 DSBs are induced only when Sγ3 is synapsed with Sμ, and that Sγ3 DSBs are then rapidly recombined with Sμ DSBs [46,47].

Despite the facts that AID-dependent *c-myc-IgH* translocations have been detected in human and mouse germinal center B cells, lymphomas, and plasmacytomas [71–73], and also in cultured activated mouse B cells with mutated DNA damage response genes [74], we did not detect AID-dependent Nbs1-binding sites in the *c-myc* locus. We were also unable to detect AID-dependent DSBs in the *c-myc* locus by LM-PCR [15]. This is consistent with the report that AID-dependent mutations per se are extremely rare (4x10^-5^ per bp) in the *c-myc* locus in germinal center B cells, except in cells lacking Ung and Msh2 where they increased by 16.8-fold [9]. These apparently conflicting results indicate that AID-induced mutations in *c-myc* are usually corrected by DNA repair [9], and only lead to detectable translocations when under selection pressure or in cells lacking DNA repair or damage response genes.

AID-dependent DSBs and translocations with I-Sce1 sites occur preferentially in super-enhancers [20,21]; super-enhancers are longer than general enhancers, are transcribed, and consist of clusters of transcription factor binding sites that regulate genes involved in cell-type specific functions [75,76]. Thus, we asked if the AID-dependent Nbs1-binding sites are located in super-enhancers, and found that although a minority of the AID-dependent and AID-independent sites are within super-enhancers, the association is highly significant (Table 1).

The RNA exosome, which degrades nascent RNA from the 3’ end when transcription is arrested, is important for allowing AID to access the transcribed DNA strand, in addition to the non-transcribed strand [77]. This would be important for forming DSBs. Recently, by the use of RNA-Seq, Pefanis et al [78] showed that transcripts initiated in the antisense direction from numerous promoters are degraded by the RNA exosome, by demonstrating that these antisense transcripts are increased in splenic B cells deficient in exosomes. They termed these exosome-dependent RNA loci *xTSS*, and found that they often correspond with regions identified by translocation capture to be AID-dependent translocation hotspots [17]. Interestingly, several of the AID-dependent DSBs detected in either of the two experiments occur at *xTSS*, and the association is highly significant (Table 1).

### What causes AID-independent DSBs?

The reproducible AID-independent Nbs1-binding sites are all in transcriptionally active regions (Table 1), and most within annotated genes (S4 Table). As discussed above, they correspond to two-ended DSBs, according to the observed positions of the strand-specific tags. Several mechanisms can generate DSBs in transcribed regions. (1) 10% of the AID-independent Nbs1 sites occur at CA repeats long enough to form Z DNA (≥50 bp). Z DNA has been shown to cause DSBs and deletions in an AID-independent manner, independent of replication, and involving NHEJ [79–81]. (2) If R loops within the genome are not removed by RNA-DNA helicase, RNaseH1, or exosome activity [78,82–84] they can lead to DSBs, perhaps due to activities of the transcription-coupled nucleotide excision repair enzymes XPF and XPG [85]. (3) Early replicating fragile sites (ERFS) (differing from common fragile sites) have recently been identified as sites where DSBs are induced early during S phase in cells undergoing replication stress in an AID-independent manner [86]. 14% of the reproducible AID-independent sites correspond to ERFS, whereas their frequency among the reproducible AID-dependent Nbs1-binding sites is not higher than random intervals (4%). (4) Topoisomerase I is known to nick transcribed regions, and recently its ability to nick DNA has been shown to be important for allowing transcription from enhancers [87]. Interestingly, SSBs introduced by Topoisomerase I can be converted to DSBs, and have been shown to bind the MRN complex.

### Conclusions

In summary, by the use of Nbs1 ChIP-Seq, we have identified hundreds of off-target AID-dependent DSBs in the genome of activated splenic B cells. More than two-thirds occur at transcriptionally active sites, as determined by RNA Pol II binding. The notable observations about these sites are (1) that ~10% of the DSBs in each experiment and 46% of the reproducible AID-dependent DSBs occur within tandem pentamer repeats ≥400 bp in length that contain WGCW motifs, the AID target hotspot. This motif creates AID hotspot targets on both strands, thus readily generating DSBs. (2) Also notable, CA repeats (≥100 bp in length) are found within ~20% of the AID-dependent DSB sites, and in 30% of reproducible sites. CA repeats form unstable Z-DNA, which could generate transient ss targets for AID; and CA repeats also increase AID-independent genome instability, perhaps due to recognition by structure specific nuclease. (3) Interestingly, Msh2 appears to contribute to DSBs at off-target sites, just as it does in the IgH S region, where it increases the conversion of SSBs induced by AID-Ung-Ape to DSBs [5]. (4) A small fraction of the DSBs appear to be generated during S phase, as they are one-ended DSBs, consistent with the finding that deficiencies in homologous recombination can increase AID-dependent genomic damage. It is also possible that some of the off-target DSBs generated during G1 phase escape into S phase, as the G1-S phase checkpoint appears to be quite weak in B cells undergoing CSR in culture [46,88]. DSBs in S phase are dangerous as they can lead to genome instability.

## Materials and Methods

### Mice

Mouse strains were extensively (≥8 generations) backcrossed to C75BL/6. AID-deficient mice were obtained from T. Honjo (Kyoto University, Kyoto, Japan) [1]. Msh2-deficient mice [89,90] were obtained from T. Mak (University Health Network, Toronto CA). Knock-out mice were always derived by breeding heterozygotes. This study was approved by, and performed in according with the guidelines provided by, the University of Massachusetts Medical School Animal Care and Use Committee. Mice were housed in a pathogen-free facility.

### B cell purification and cultures

Mouse splenic B cells were isolated and induced to switch for two days to IgG3 as previously described [46].

### Retroviral constructs and virus production

pMX-PIE-AID-FLAG-ER-IRES-GFP-*puro* [44] was received from Drs V. Barreto and M. Nussenzweig (The Rockefeller University, NY). The control retrovirus pMX-PIE-ER-IRES-GFP was previously described [91]. Production of viruses and infection of B cells was previously described [91].

### LM-PCR and ChIP

Genomic DNA preparation, LM-PCR, and quantitative ChIP were performed as described [46]. Antibodies for ChIP were: Nbs1 (Abcam, ab32074), RNA Pol II (Millipore, 04–1572), and ER (Santa Cruz Biotechnology sc-8002X). Primers used for LM-PCR are listed in S5 Table. Three-fold more template DNA was used in each lane of the LM-PCR gel to examine off-target DSBs compared with that used for Sμ DSBs.

### ChIP-Seq analysis

A modified version of the Illumina protocol was followed to prepare ChIP DNA samples for the deep sequencing pipeline. Briefly, blunting of the fragments was performed using the END-IT DNA repair kit (Epicentre) followed by the addition of a dA overhang using exo-minus Klenow (Epicentre). Paired-end adapters (Illumina) were ligated using the fast link kit (Epicentre). The fragments were amplified twice using the Illumina PE primers and PfuUltra II Fusion HS DNA polymerase (Stratagene), and each round of PCR was followed by gel purification and sizing of the fragments. Samples were cloned using the Topo cloning system (Invitrogen) and several clones were sequenced to assess sample quality prior to submission for sequencing on the Illumina GAII (Exp 1) or HiSeq 2000 (Exp 2) platforms at the UMASS Deep Sequencing Core facility, obtaining either 36 bp single-end (Exp 1) or 50 bp paired-end reads (Exp 2 and Pol2).

#### Overview of bioinformatic analyses

Sequences were aligned to the mouse mm9 reference genome, retaining only unique alignments. After duplicate removal, total reads were: Exp 1 WT, 6,031,566; Exp 1 *aid* ^*-/-*^, 17,620,060; Exp 2 WT, 12,068,745; Exp 2 *aid* ^*-/-*^, 19,214,464. Initial peak calling for Nbs1 ChIP’s was by the Homer findPeaks program [43] using *aid* ^*-/-*^ ChIP reads as the control. The resulting peak lists were inspected on the IGV genome browser [92] to establish additional filtering thresholds based on total tag counts and signal/noise. The Homer mergePeaks program was used for peak intersection and annotation. Co-occurrence statistics were obtained using the IntersectRegions program of the USeq suite [93]. Tandem repeats were identified by Tandem Repeat Finder [94], and original Perl scripts were executed to parse the output to determine the WGCW content of the identified tandem repeats. Peaks were called from the Pol II ChIP (14,594,564 total aligned reads) using SICER [95].

#### Detailed bioinformatics methods

Initial alignment of ChIP sequence reads to the mouse mm9 reference genome (NCBI37) was by ELAND as part of the Illumina CASAVA pipeline. Unaligned reads were subsequently aligned by Bowtie (v1.0.0) using the options-n2-strata, accepting only unique mappings (-m1), and combined with the ELAND alignments. Due to the small size of the Exp 1 WT Nbs1 ChIP library, all duplicate mappings were removed from both WT and *aid* ^*-/-*^ alignments. Two duplicates were retained for both Exp 2 libraries.

Nbs1 peak calling was performed using Homer (http://homer.salk.edu/homer/) findPeaks in factor mode, using fragment lengths estimated by the makeTagDirectory program. FindPeaks was run with the corresponding *aid* ^*-/-*^ library as control using a window size of 500 bp and a fold change threshold of 2.0. An empirical filtering scheme based on total peak tag counts was applied to eliminate questionable calls, as determined by viewing read coverage tracks on the IGV genome browser. The filtering thresholds for Exp 1 were (tag count, WT: *aid* ^*-/-*^ threshold, WT: local background threshold). If ≥18 tags, then ≥2.0 or ≥6.0; if 17–16 tags, then ≥2.5 and ≥4.0; if 15–14 tags, then ≥3.6 and ≥4.0; if 13 tags, then ≥9.0 and ≥8.0. For Exp2, the thresholds were: >52 tags, then ≥2.2 or ≥6.0; if 52–22 tags, then ≥2.2 and ≥6.0; if 21–19 tags, then ≥4.0 and ≥10.0. For subsequent downstream analyses, the peak coordinates were extended 1000 bp from the center. AID-independent Nbs1 binding sites were obtained by running findPeaks with 500-bp windows and default parameters using the WT Nbs1 ChIP tags as input but no control. The resulting Nbs1-enriched (vs. local background) sites were then filtered to remove intervals having a WT Nbs1: *aid* ^*-/-*^ tag count ratio greater than 1.4. The respective tag counts were obtained using Homer annotatePeaks. RNA pol II peaks were called by SICER v1.1 using the parameters W200, G600, E1000 (Zang et al., 2009). Transcribed genes were identified by intersecting UCSC known gene transcripts with the RNA pol II peaks. Coverage tracks were generated by the ReadCoverage program of the USeq suite [93], after first extending the reads to the estimated fragment length. Stranded coverage tracks were obtained using the Homer makeUCSCfile program.

Nbs1 peak intersections reported in Tables 1 and S1–S4 were obtained using Homer mergePeaks. Target intervals were downloaded from the supplementary data tables of the publications cited in Table 1. Statistical significance was assessed using the USeq IntersectRegions program, which compares the observed result to that of 1000 randomization trials in which random chromosome intervals matched to the target set (length and chromosome distribution) are used. One-ended break sites were identified by strand-biased read counts over the Nbs1 binding interval. Aligned reads from the WT Nbs1 ChIP’s were separated by strand, and summed separately for each for Nbs1 peak. Peaks having a strand bias ≥ 2.8-fold in either direction were defined as one-ended. For intersections with AID-dependent Nbs1-sites identified by ChIP-chip [15], the ChIP-chip peak intervals (mm8) were converted to mm9 using the LiftOver tool at http://genome.ucsc.edu/cgi-bin/hgLiftOver.

Tandem repeats within Nbs1 binding sites were found using Tandem Repeat Finder [94]. Default parameters were used except for minimum score, which was set to 100 and 60 for WGCW and CA repeats, respectively. Identified repeats containing ≥ 90% CA/TG in the core repeat motif were classified as CA. WGCW occurrences within identified repeat regions were counted using EMBOSS fuzznuc [96]. Genome averages of CA and WGCW repeats were estimated by running the Tandem Repeat Finder analysis on 10,000 random genomic intervals matched to the length and chromosome distribution of the combined WT Nbs1 peak sets. Repeat sequences having ≥ 2.0 occurrences of WGCW per 100 bp were classified as WGCW repeats; random genomic repeats identified by Tandem Repeat Finder are relatively low in WGCW content (average is 0.673 occurrences/100 bp tandem repeat).

ChIP-seq data have been deposited into the GEO database. Series accession **#** GSE66424.

http://www.ncbi.nlm.nih.gov/geo/query/acc.cgi?acc=GSE66424.

## Supporting Information

S1 Table AID-dependent Nbs1-binding sites identified in Exp 1. (XLSX)

S2 Table AID-dependent Nbs1-binding sites identified in Exp 2. (XLSX)

S3 Table Reproducible AID-dependent Nbs1-binding sites. (XLSX)

S4 Table Reproducible AID-independent Nbs1-binding sites. (XLSX)

S5 Table Primers used in LM-PCR experiments. (XLSX)

S1 Fig Reproducible AID-dependent DSBs on chromosome 8. Intergenic site that lacks Pol II binding, WGCW, and CA tandem repeats. A. Browser tracks. B. LM-PCR of site demonstrates that DSBs are AID-dependent. This site was not tested for Msh2-dependence.
(PDF)

S2 Fig Reproducible AID-dependent DSBs on chromosome 17. Intergenic site that shows Pol II binding, but lacks WGCW and CA tandem repeats. A. Browser tracks. B. LM-PCR demonstrates that DSBs at site are AID and Msh2-dependent.
(PDF)

S3 Fig Reproducible AID-dependent DSBs on chromosome 17. This site considered reproducible because when the sites in the individual experiments are extended by 1 kb from their center, the intervals overlap. Intergenic site that has Pol II binding and WGCW tandem repeats, but not CA repeats. A. Browser tracks. B. LM-PCR demonstrates that DSBs at site are AID and Msh2-dependent.
(PDF)

S4 Fig AID-dependent DSBs on chromosome 5 called in Exp 1 but not Exp 2. Site is located in the *hip1* gene, shows Pol II binding, but lacks WGCW and CA tandem repeats. A. Browser tracks. B. LM-PCR demonstrates that DSBs at site are AID and Msh2-dependent.
(PDF)

S5 Fig Strand bias of aligned tags from Nbs1 and Pol II ChIPs. About 6% (see Table 1) of Nbs1-binding sites exhibit strand bias consistent with being one-ended DSBs. Shown are frequency distributions of log_2_-transformed ratios of plus strand over minus strand tag counts for Nbs1- and Pol2-binding sites. Sites with strand bias absolute log_2_ ratios > 1.5 (indicated by blue vertical lines) have 2.8 fold more signal on one strand than the other and are defined here as one-ended DSBs.
(EPS)

S6 Fig Correspondence between AID-dependent Nbs1 ChIP-seq and ChIP-chip [15] sites on chromosome 2. Shown below the ChIP-Seq results are 4 panels from the two Nbs1 ChIP-chip experiments showing NimbleScan FindPeaks calls from WT and *aid* ^*-/-*^ cells.
(EPS)

S7 Fig Correspondence between AID-dependent Nbs1 ChIP-seq and ChIP-chip [15] sites on chromosome 10. Shown below the ChIP-Seq results are 4 panels from the two Nbs1 ChIP-chip experiments showing NimbleScan FindPeaks calls from WT and *aid* ^*-/-*^ cells.
(EPS)
