# Supplementary material for: Nbs1 ChIP-Seq Identifies Off-Target DNA Double-Strand Breaks Induced by AID in Activated Splenic B Cells
Source: PLoS Genet. 2015 Aug 11;11(8):e1005438. doi: 10.1371/journal.pgen.1005438 (PMC4532491; doi:10.1371/journal.pgen.1005438)
Supplement: S1 Fig — Intergenic site that lacks Pol II binding, WGCW, and CA tandem repeats. A. Browser tracks. B. LM-PCR of site demonstrates that DSBs are AID-dependent. This site was not tested for Msh2-dependence. (PDF) [file pgen.1005438.s006.pdf]

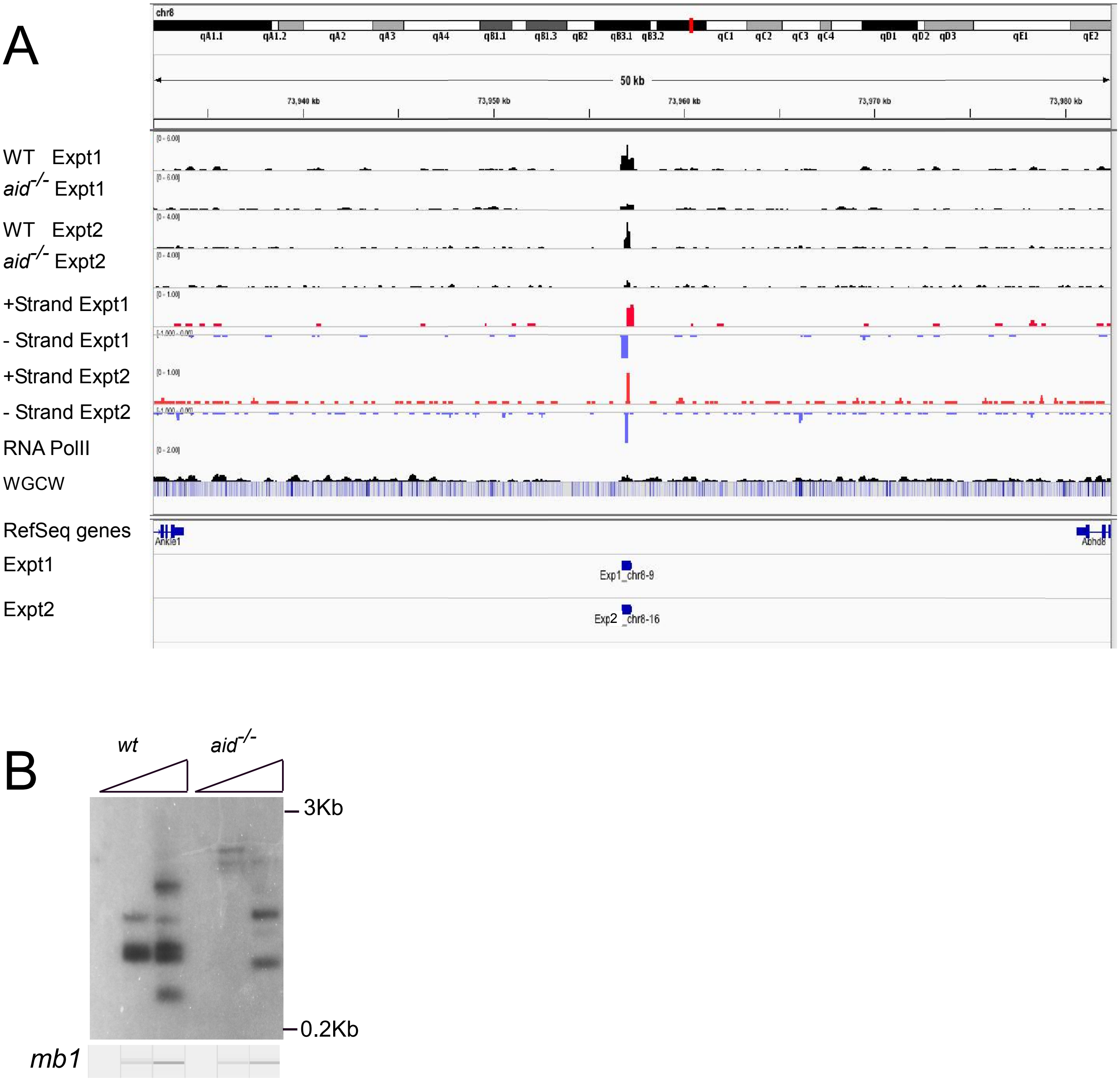

A
chr8
qA1.1
qA1.2
qA2
qA3
qA4
qB1.1
qB1.3
qB2
qB3.1
qB3.2
qC1
qC2
qC3
qC4
qD1
qD2
qD3
qE1
qE2
50 kb
73,940 kb
73,950 kb
73,960 kb
73,970 kb
73,980 kb
WT Expt1
aid-/- Expt1
WT Expt2
aid-/- Expt2
+Strand Expt1
- Strand Expt1
+Strand Expt2
- Strand Expt2
RNA PolII
WGCW
RefSeq genes
Expt1
Expt2
Ankle1
Abhd8
Exp1_chr8-9
Exp2_chr8-16
B
wt
aid-/-
3Kb
0.2Kb
mb1
