# Supplementary material for: Nbs1 ChIP-Seq Identifies Off-Target DNA Double-Strand Breaks Induced by AID in Activated Splenic B Cells
Source: PLoS Genet. 2015 Aug 11;11(8):e1005438. doi: 10.1371/journal.pgen.1005438 (PMC4532491; doi:10.1371/journal.pgen.1005438)
Supplement: S2 Fig — Intergenic site that shows Pol II binding, but lacks WGCW and CA tandem repeats. A. Browser tracks. B. LM-PCR demonstrates that DSBs at site are AID and Msh2-dependent. (PDF) [file pgen.1005438.s007.pdf]

A

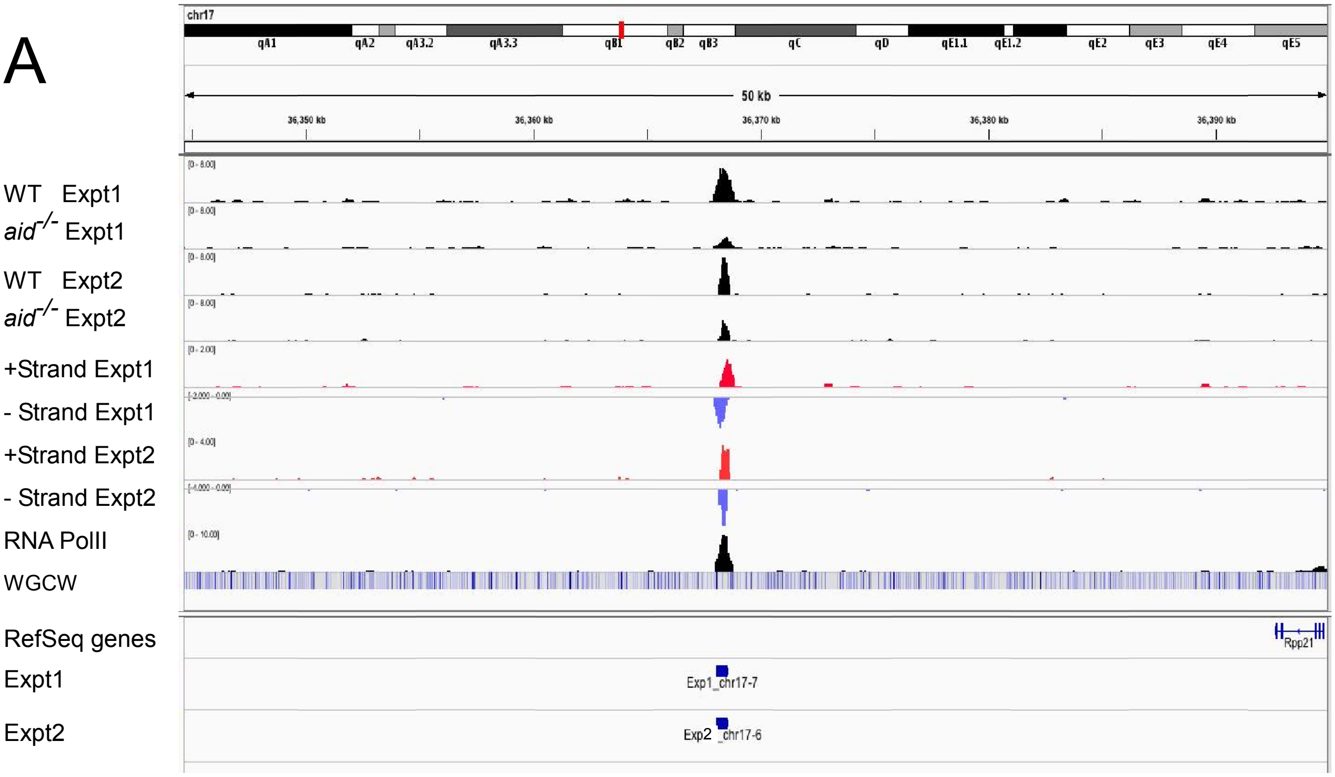

chr17
qA1
qA2
qA3.2
qA3.3
qB1
qB2
qB3
qC
qD
qE1.1
qE1.2
qE2
qE3
qE4
qE5
50 kb
36,350 kb
36,360 kb
36,370 kb
36,380 kb
36,390 kb
WT Expt1
aid-/- Expt1
WT Expt2
aid-/- Expt2
+Strand Expt1
- Strand Expt1
+Strand Expt2
- Strand Expt2
RNA PolII
WGCW
RefSeq genes
Rpp21
Expt1
Exp1_chr17-7
Expt2
Exp2_chr17-6

B

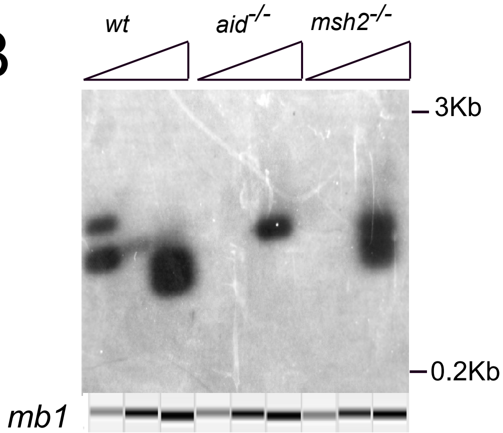

wt
aid-/-
msh2-/-
3Kb
0.2Kb
mb1
