# Supplementary figures and images for: Nbs1 ChIP-Seq Identifies Off-Target DNA Double-Strand Breaks Induced by AID in Activated Splenic B Cells
Source: PLoS Genet. 2015 Aug 11;11(8):e1005438. doi: 10.1371/journal.pgen.1005438 (PMC4532491; doi:10.1371/journal.pgen.1005438)

A

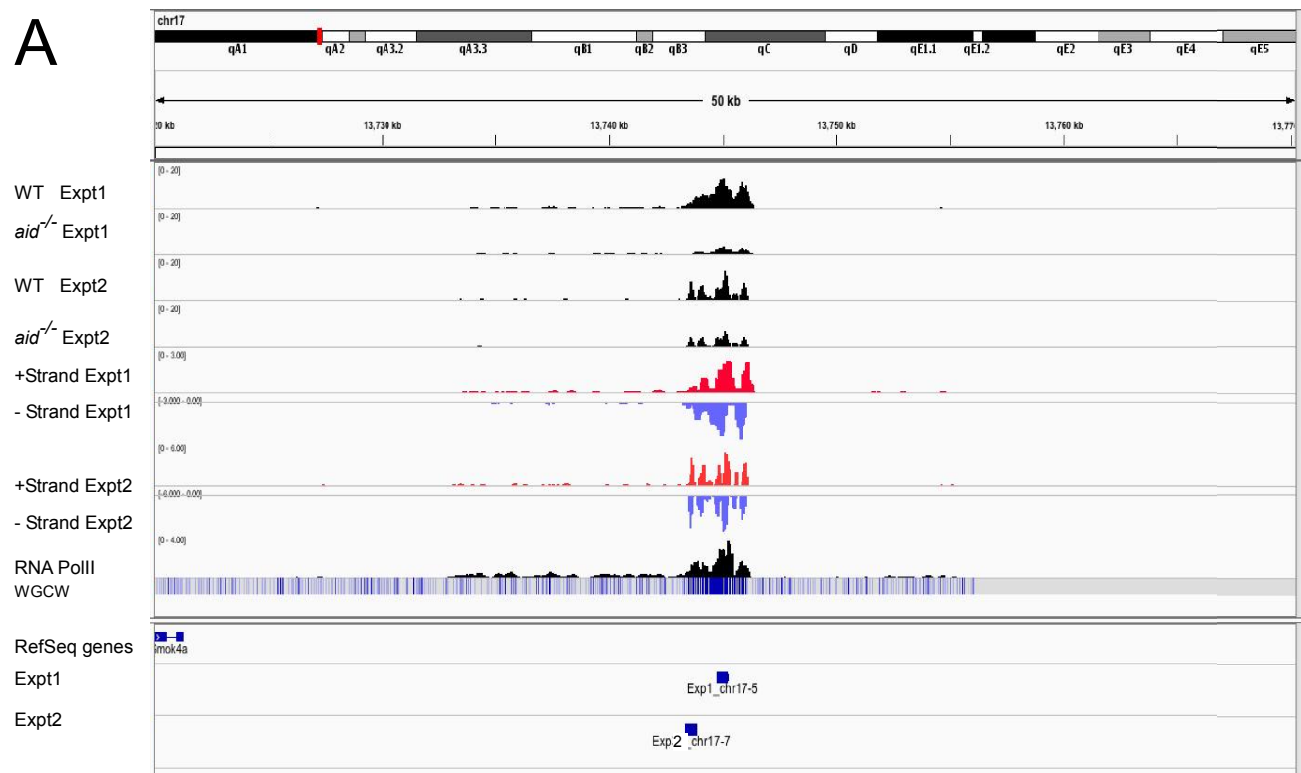

B

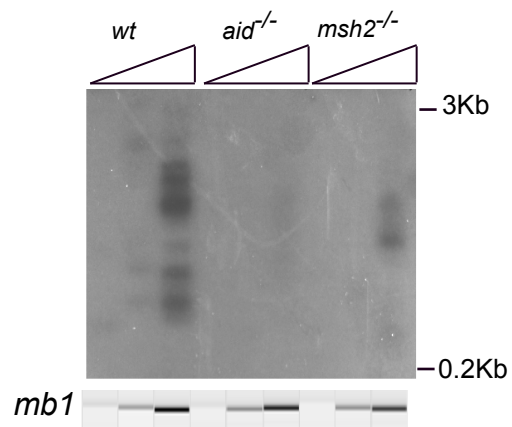

Supplement: S3 Fig — This site considered reproducible because when the sites in the individual experiments are extended by 1 kb from their center, the intervals overlap. Intergenic site that has Pol II binding and WGCW tandem repeats, but not CA repeats. A. Browser tracks. B. LM-PCR demonstrates that DSBs at site are AID and Msh2-dependent. (PDF) [file pgen.1005438.s008.pdf]
