# Supplementary material for: Nbs1 ChIP-Seq Identifies Off-Target DNA Double-Strand Breaks Induced by AID in Activated Splenic B Cells
Source: PLoS Genet. 2015 Aug 11;11(8):e1005438. doi: 10.1371/journal.pgen.1005438 (PMC4532491; doi:10.1371/journal.pgen.1005438)
Supplement: S4 Fig — Site is located in the hip1 gene, shows Pol II binding, but lacks WGCW and CA tandem repeats. A. Browser tracks. B. LM-PCR demonstrates that DSBs at site are AID and Msh2-dependent. (PDF) [file pgen.1005438.s009.pdf]

A

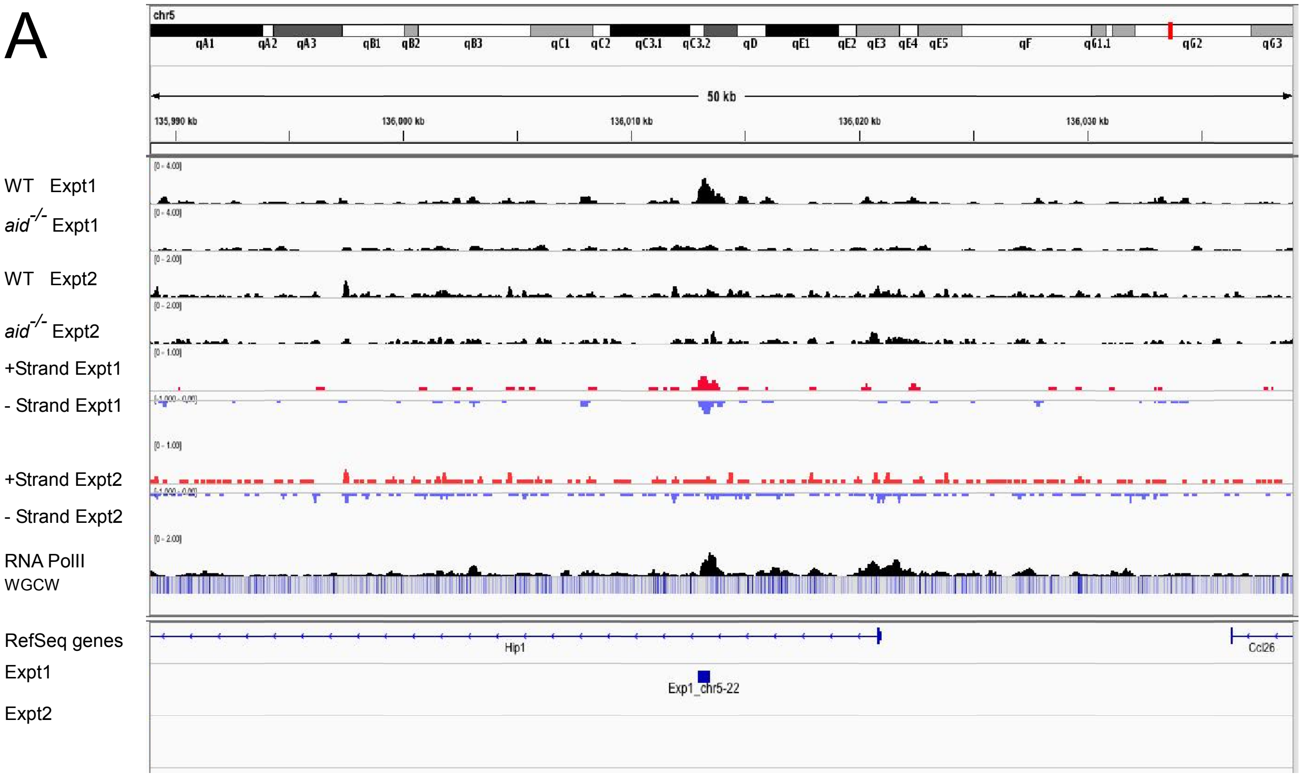

chr5
qA1
qA2
qA3
qB1
qB2
qB3
qC1
qC2
qC3.1
qC3.2
qD
qE1
qE2
qE3
qE4
qE5
qF
qG1.1
qG2
qG3
50 kb
135,990 kb
136,000 kb
136,010 kb
136,020 kb
136,030 kb
WT Expt1
aid-/- Expt1
WT Expt2
aid-/- Expt2
+Strand Expt1
- Strand Expt1
+Strand Expt2
- Strand Expt2
RNA PolII
WGCW
RefSeq genes
Expt1
Expt2
Hip1
Ccl26
Exp1_chr5-22

B

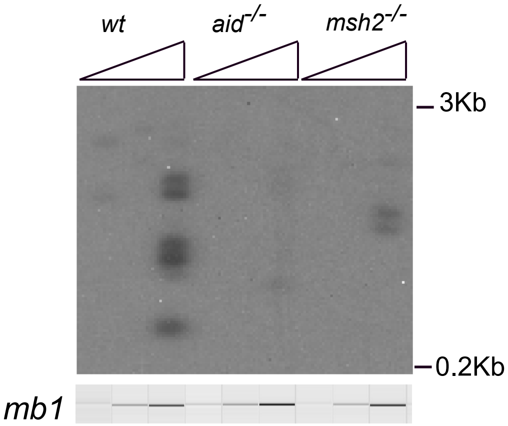

wt
aid-/-
msh2-/-
3Kb
0.2Kb
mb1
